# Multifocal optical coherence tomography of the mouse eye to image the vitreoretinal vasculature in full depth

**DOI:** 10.1117/1.JBO.30.11.116002

**Published:** 2025-11-03

**Authors:** Simon Brais-Brunet, Raphaël Maltais-Tariant, Caroline Boudoux, Mathieu Dehaes

**Affiliations:** a Université de Montréal, Institute of Biomedical Engineering, Montreal, Quebec, Canada; b Centre de Recherche Azrieli du CHU Sainte-Justine, Montreal, Quebec, Canada; c Polytechnique Montréal, Department of Engineering Physics, Montreal, Quebec, Canada; d Castor Optics, Saint-Laurent, Quebec, Canada; e Université de Montréal, Department of Radiology, Radio-Oncology and Nuclear Medicine, Montreal, Quebec, Canada

**Keywords:** optical coherence tomography, multifocal imaging, animal model, vitreoretinal vasculature, hyaloid, retina

## Abstract

**Significance:**

*In vivo* optical coherence tomography (OCT) of the mouse vitreoretinal vasculature in full depth is technically challenging. Conventional OCT techniques employ axial confocal gating, which induces signal drop-off and limits spatial resolution outside the Rayleigh range.

**Aim:**

Our aim is to develop a multifocal OCT imaging approach using a tunable lens and a registration method that allows the generation of a composite image of the vitreoretinal vasculature while preserving high and uniform lateral spatial resolution, signal intensity, and image contrast in full depth.

**Approach:**

A calibration target was developed to characterize the multifocal optical system and quantify the signal intensity, contrast, and resolution. These optical specifications were used to image mice at postnatal day 14. Intra- and inter-volume registration methods were necessary to correct for motion and generate a composite image from single-focus images using weighted averaging.

**Results:**

In the calibration target, signal intensity and contrast were 20 dB higher in the composite compared with single-focus images. Lateral resolution remained uniform (4 to 6  μm). In animals, signal intensity and contrast were 10 to 15 dB higher in the composite compared with single-focus images and highest in the hyaloid vasculature.

**Conclusions:**

This technique is promising in studying the mouse vitreoretinal vasculature during eye development and disease.

## 1 Introduction

Animal models are frequently used to study the vasculature of the eye in the context of physiological development and disease.^1^^–^^4^ During retinal development, pathological conditions can affect the growth of blood vessels located in the retina and vitreous.^5^^–^^11^ In the vitreous, the hyaloid vasculature plays a critical role in the development of anatomical structures, providing oxygen and nutrients to the crystalline lens and other ocular tissues.^1^^,^^12^^,^^13^ Observing the development of this vasculature is critical to understanding the pathogenesis of vascular ocular diseases such as retinopathy of prematurity.^6^^,^^7^^,^^14^ Methodological approaches based on the concurrent *in vivo* characterization of blood vessels located in the retina and vitreous (i.e., the vitreoretinal vasculature) are necessary to study the interconnections of these vascular systems during eye development and disease.

Optical coherence tomography (OCT) has previously been used for *in vivo* imaging of the hyaloid vasculature in the context of vascular vitreoretinopathy in a rat model.^2^^,^^10^^,^^11^^,^^15^ In a previous study, imaging the hyaloid and retinal vasculature with optimal resolving power required two imaging sessions, one for each structure.^11^ Following data acquisition sessions, OCT volumes were combined to obtain a structural description of vascular vitreoretinopathy. This optomechanical approach was based on the modification of the location of the ocular lens in the optical path to change the focal plane position.^16^ However, this operation modifies the position of the galvanometric mirror conjugate plane, generally located at the eye pupil, which induces significant changes in the field curvature. This, in turn, requires the animal to be repositioned to minimize image distortion. This procedure may affect image registration and fail to generate a composite image of the full field of view. In the mouse, such manipulations are even more critical due to the smaller size of the eye and its higher curvature, compared with that of the rat. The mouse model remains the most prevalent mammal model, and adapting imaging techniques to mouse eye size is relevant.^3^^,^^17^

In addition, this approach mirrors a strategy in which the scanning head translates along the OCT beam axis.^18^ This configuration allowed for synchronizing the OCT confocal gate with its coherence gate, dynamically changing the position of the beam waist in a time-domain OCT scan. However, data acquisition using this configuration is too slow in the context of *in vivo* mouse retinal imaging.

Another approach involved adjusting the beam vergence at the location of the eye pupil without using manual displacements of the ocular lens.^19^ In this optical configuration, the distance between the source fiber and the collimator is modified, which allows the focal plane locations to be selected without distortion of the field curvature. This method is time-consuming and unsuitable for multifocal imaging, which requires image registration to generate a composite image of the full field of view.

A focus-tunable lens placed in the optical path, ahead of the galvanometric mirrors, can solve the previously mentioned issues and rapidly adjust the position of the focal plane within the tissue of interest. This instrumentation was previously used for brain imaging to generate composite images showing a signal distribution that was not altered by the confocal gate of the optical system.^20^ Although the signal intensity was characterized, the resolving power of the system was not quantified.

A tunable lens was previously used to precisely adjust the focal plane in a specific retinal layer, compensating for refractive errors and optimizing spatial resolution.^21^^–^^23^ A tunable lens was also used to study the anterior part of the human eye^24^ and the retina.^25^ The combination of a tunable lens with the Fourier transform mirror image^26^ or a dual-band OCT^27^ allowed quasi-simultaneous imaging of the two structures. However, the prior technique is not suitable when scattering material, such as hyaloid vessels, is present in the vitreous, whereas the latter requires a second OCT system. A system based on an acousto-optic tunable lens was also previously used for this purpose, which was faster and more robust to chromatic aberration.^28^ Although the benefits of this technique are important, its implementation is complex and requires synchronized light modulation and a custom-made acousto-optic device.

The feasibility of using a tunable lens to image the mouse vitreoretinal vasculature at multiple focal planes and the combination of these images to generate a composite image of the full field of view using a registration method remains to be demonstrated. The objective of this study was to develop a multifocal OCT imaging approach using a tunable lens and a registration method that allowed the generation of a composite image of the vitreoretinal vasculature while preserving high and uniform lateral spatial resolution, signal intensity, and image contrast throughout the full depth. This technique was characterized using a custom-made calibration target, and its feasibility was demonstrated in mice through *in situ* and *in vivo* experiments.

## 2 Material and Methods

### 2.1 Imaging System

**Fig. 1:**
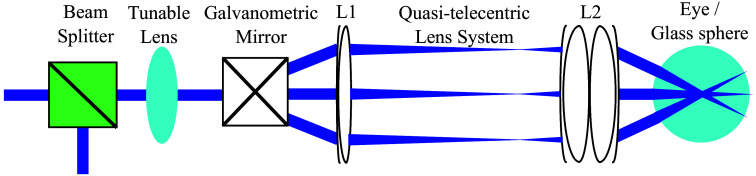
Schematic of the optical configuration used for angular scanning in the calibration target and animals. Lenses L1 and L2 were selected to maximize light entering the mouse eye or the glass sphere.

OCT images were acquired with a spectral-domain system (Telesto 220, Thorlabs, Newton, New Jersey, United States) that emits light at 1300 nm. The nominal axial pixel size of the system is 3.5  μm in the air, whereas 2.6  μm in the retina, considering a refractive index of n∼ 1.33. The optical resolution is 5.5  μm in the air and 4.1  μm in the retina. The use of a customizable scanner head (OCTP-1300/M, Thorlabs, Newton, New Jersey, United States) and scanning lens (see Sec. 2.3) allowed the addition of a tunable lens (EL-03-10-NIR, Optotune, Dietikon, Switzerland) to the optical path. The electrically tunable lens was selected for its wide range, varying from −13 to +13 diopters. Positioning the lens between the beam splitter and the galvanometers allowed a displacement of the focal plane anterior to the retina without shifting the galvanometric mirror conjugated plane located in the pupil (Fig. 1). This configuration was designed to minimize field of view distortion (see Fig. S1 in the Supplementary Material) while acquiring high-resolution images at multiple depths of focus. These multifocal OCT images were further registered to generate a composite image with an arbitrarily large depth of focus.

### 2.2 Calibration Target

**Fig. 2:**
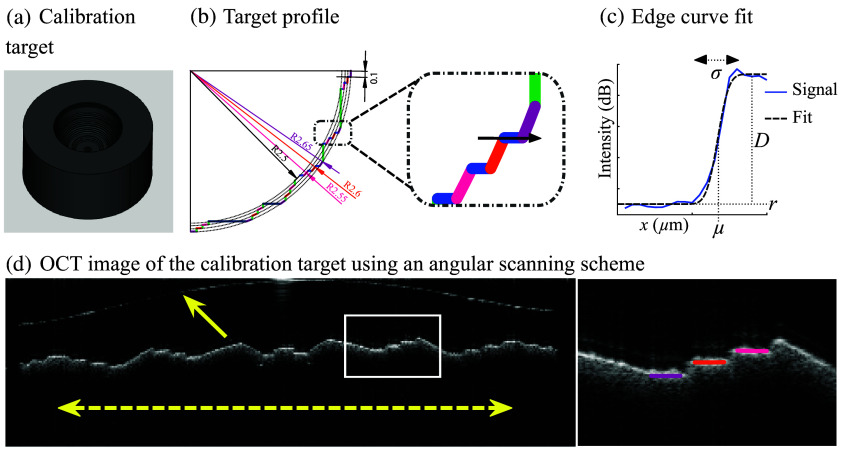
(a) Quantification of the spatial resolution of the image using the calibration target. (b) The target was engraved with spherical (R, θ) and Cartesian (x,y) structures. In the zoomed image, three spherical structures with specific radii are shown (R=2.55  mm in magenta, R=2.60  mm in orange, and R=2.65  mm in violet) as well as Cartesian structures (x in blue and y in green). The signal intensity (dB) measured along the black arrow in sub-figure (b) is shown in sub-figure (c) as a function of lateral position x (μm, blue line). The signal is overlapped with the fitted error function (black dashed line): the fitting process extracts μ and σ, representing the midpoint and the width of the interface between the background residue and the intensity of the structure, respectively. The metric σ was further used to characterize the lateral spatial resolution of the image. (d) An OCT image acquired in the calibration target with the glass sphere mimicking the eye and using the angular scanning scheme. The solid yellow arrow indicates the surface of the glass sphere. The yellow dashed double arrow shows the spatial range of a scan of 90 deg or ∼3.927  mm. Engraved structures located in the white box are magnified in the right sub-figure. The radial structures were 100  μm long and vertically spaced by 50  μm from one to another.

A hemispherical calibration target was developed on a diamond turning lathe to characterize the optical system and quantify the signal intensity, image contrast, and lateral spatial resolution at several depths of focus. The target was engraved with spherical (R, θ) and Cartesian (x,y) structures (Fig. 2). In the zoomed image of Fig. 2(b), three spherical structures with specific radii are shown (i.e., R=2.55  mm in magenta, R=2.60 mm in orange, and R=2.65  mm in violet) as well as Cartesian structures (x in blue and y in green). The radial structures were 100  μm long and vertically spaced by 50  μm from one to another. In addition to the hemispherical target, a complementary 5 mm diameter glass sphere (SLAH-79 ball lens, Edmund Optics, Barrington, New Jersey, United States) was used to mimic an eye the size of a mouse and facilitate optical alignment for *in vivo* experiments. Using the high refractive index of the glass (n∼ 2 at 1300 nm), the collimated beam entering the sphere was focused on its opposite side, mimicking the function of the eye from the pupil to the retina.

The combination of the calibration target and glass sphere allowed for the evaluation of the pixel-to-distance ratio and the pixel-to-angle ratio in the lateral and angular scanning schemes, respectively [metrological calibration, Fig. 2(d)]. Angles were further converted to distances through the axial length of the imaged eye/glass sphere. The pixel-to-distance ratio was then applied to animal experiments.

### 2.3 Optical Imaging Configuration and Data Acquisition

Volumetric OCT images of the calibration target (without the glass sphere) were acquired with a scan lens (LSM03, Thorlabs, Newton, New Jersey, United States) of an effective focal length of 36 mm. The known optical specifications of the LSM03 lens allowed the characterization of the spatial resolution when varying the tunable lens electrical current. The scanning pattern was a 512×512×1024  pixels cube (fast axis, slow axis, depth) that covered a $6\;{\mathrm{mm}}^{2}$ area with 3.5 mm depth (in air). Five focal planes were defined at equidistant depths to characterize the optical system with the calibration target. The five focal ranges were centered at 0.26 mm (focus #1), 0.96 mm (focus #2), 1.65 mm (focus #3), 2.35 mm (focus #4), and 3.05 mm (focus #5). Animal imaging required the use of an optical relay based on the combination of two lenses: L1, an achromatic doublet lens (AC-254-150-C, Thorlabs, Newton, New Jersey, United States) of an effective focal length of f=150  mm and L2, a lens composed of two achromatic doublets (AC-254-030-C, Thorlabs, Newton, New Jersey, United States) of a combined focal length of f∼15  mm (Fig. 1).^29^ This configuration was used to reduce the beam size and optimize light entering the pupil to image the vitreoretinal space. Two types of data were acquired from the animals. For the first dataset, 100 coplanar B-scans were acquired by varying the electrical current of the tunable lens from 0 to 6 mA. This approach allowed the calibration of the electrical current sent to the tunable lens to focus on a specific feature. For the second dataset, three focal planes were selected and located in the crystalline lens, the anterior retina, and the posterior retina/choroid, respectively. For each focal plane, C-scans were acquired and centered around the optic nerve.

### 2.4 Data Processing

**Fig. 3:**
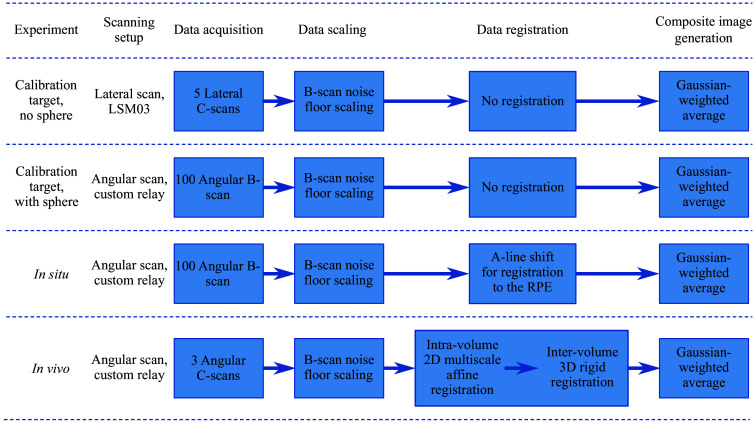
Flowchart of data processing and composite image generation for each experiment. RPE, retinal pigment epithelium.

The data processing steps used for each experiment are described in Fig. 3 and detailed in this section.

#### 2.4.1 Data scaling

Uniform data scaling was necessary to quantitatively compare images acquired in the calibration target and animals and to combine multifocal images in a composite image. The image scale is typically defined by its maximum range, which creates a bias from one image to another as the maximum and minimum intensity vary between two data acquisitions. To overcome this limitation, the scaling factor was defined by the median (med(·)) of the raw signal intensity I, representing the noise floor, which remained relatively stable between data acquisition sessions. The scaled signal (S) was given by

$$S=\frac{I-\mathrm{med}(I)}{\mathrm{med}(I)}.$$ (1)

Values of S below 0 were approximated to 0. Due to the nonuniform OCT background noise distribution, where the background noise is slightly higher at z=0, the subtraction of med(I) in Eq. 1 may induce a nonzero background residue (r, see details in Sec. 2.5.3). An example of the scaling technique applied to OCT images is provided in Fig. S2 of the Supplementary Material.

#### 2.4.2 Intra-volume registration

Intra-volume registration was not necessary for data acquired in the calibration target. Otherwise, two data registration schemes were used for data acquired in animals. For the first dataset (100 angular and coplanar B-scans), the retinal pigment epithelium (RPE) was used as a reference. It was first detected and segmented using a previously published algorithm.^30^ The RPE is a useful reference for registration as it is the most reflective structure of the retina when using an OCT that emits light at 1300 nm. In this registration scheme, the A-lines were vertically shifted. The retina became flat after registration due to this shift process. Due to the absence of bulk motion in this dataset (see Sec. 2.6), this registration scheme was sufficient to identify the function between the electrical current of the tunable lens and the focal depth within the eye. For the second dataset (three angular C-scans), a multiscale frame-by-frame affine registration method was used to correct for bulk motion (e.g., heart and respiration rates) along the slow axis.^31^ Registration was initiated at the central B-scan, which was used as the first reference. From that frame, the newly registered B-scan was used as a reference for registration of the next B-scan. The registered volumes were visually inspected, and the frames that induced significant propagation of errors were removed from the original volume. In these cases, registration was reapplied to the filtered volume. Along the slow axis, the registration process eliminated the apparent curvature of the retina, which also resulted in a flattened retina. Volumes were also registered along the fast axis, resulting in a flattened retina in both lateral orientations. Therefore, retina flattening was an unexpected (but beneficial) consequence of this second image registration scheme.

#### 2.4.3 Inter-volume registration and composite image

To correct a potential spatial drift between data acquired at specific focal depths, a 3D rigid registration process, typically used for stitching, was applied to *in vivo* OCT volumes.^32^^,^^33^ For all experiments, the composite image was generated by combining the focal range of each dataset using a weighted averaging approach similar to that of a previous study.^20^ Each component of the composite image was generated by the maximum intensity projection (MIP) of raw data, which were weighted by a Gaussian curve centered in the middle of the focal range. The distance between each focal transition was defined by the full-width-half-maximum (FWHM) of each Gaussian-weighted curve. This distance ensured that the centers of successive Gaussian curves were separated by their FWHM value.

### 2.5 Image Metrics

#### 2.5.1 Signal intensity

Scaled signal intensity (S) was visually and quantitatively compared between single-focus and composite images for the three experiments. The composite image intensity range comprised the maximum intensity of all registered single-focus volumes.

#### 2.5.2 Image contrast

Comparison of image contrast between single-focus and composite images was performed by transforming each image to a single-dimensional vector along the depth axis using an MIP. For the calibration target, a 2D section along the fast axis intersecting the center of the hemispherical target was extracted. For 2D images acquired in animals, an MIP was applied along the fast axis. For 3D images acquired in animals, an MIP was applied along the slow and fast axes, respectively. A contrast-to-noise ratio (${\mathrm{CNR}}_{S}$) of the scaled signal S [from Eq. (1)] was defined such that

$${\mathrm{CNR}}_{S}=\frac{S}{\mathrm{var}(S_{\Omega })},$$ (2)

where var($S_{\Omega }$) is the noise variance assessed in a region Ω of the composite image where there is no OCT reflectance (S<15.45  dB, when combining the three experiments).

#### 2.5.3 Lateral spatial resolution

In the calibration target, three regions-of-interest (ROIs), including horizontal plateau structures [zoomed image in Fig. 2(b)], were selected for lateral edge analysis. ROIs were located at specific depths: 1, 1.5, and 2 mm from the top of the calibration target. To quantify lateral spatial resolution, a function g defined by the cumulative distribution function of a Gaussian curve was fitted to the experimental data, such that^34^

$$g(x)=D\cdot [1+\mathrm{erf}(\frac{x-\mu }{\sigma \sqrt{2}})]+r,$$ (3)

where D is the difference between the maximum signal intensity and the background residue r [Fig. 2(c)]. This fitting process was performed to extract the mean μ and variance σ of the *error function* (erf), which described the midpoint and width of the interface, respectively. At μ±1.645σ, signal intensity reached 10% and 90% of D, respectively, thus mimicking the 10% to 90% distance method to quantify the resolution of an image. The metric σ was further used to characterize the spatial resolution of the image. The performance of the fit was assessed by the coefficient of determination ($R^{2}$). The *complementary error function* (erfc(x)=1−erf(x)) was used in Eq. (3) instead of erf(x) when an artifact on the left side of the structure biased the evaluation of the background residue r.

### 2.6 Animals

*In vivo* images were acquired in two mice that were bred locally (129S1/SvImJ, Jackson Laboratory, Bar Harbor, Maine, United States) at postnatal day 14 (P14) and according to the Animal Care Committee of the *Centre de Recherche Azrieli du CHU Sainte-Justine* (Montreal, Canada). P14 was selected because the eyelids typically open at this age. After anesthesia was performed with a mixture of ketamine (Vetoquinol, 100 mg/kg) and xylazine (Bayer, 20 mg/kg) at a dose of 10  μL/g phenylephrine drops (Alcon, 2.5%) were used to keep the pupil dilated during imaging. Teardrops were applied to the cornea to prevent corneal dryness. After image acquisition, the animal was sacrificed while asleep using a pentobarbital solution (Euthanyl, Bimeda-MTC). The death was further confirmed with cervical dislocation. However, the death of one animal was observed during data acquisition at the end of a set of B-scans. In this case, the final procedures of the sacrifice protocol were administered according to institutional ethical practice. B-scans were recorded, and this experiment was defined as *in situ*.

## 3 Results

### 3.1 System Characterization: Calibration Target

**Fig. 4:**
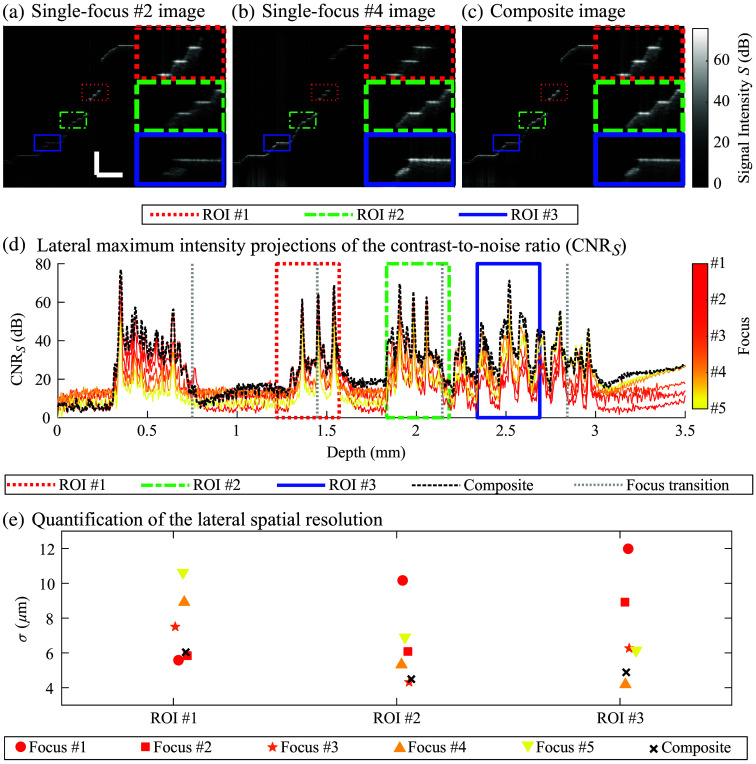
OCT imaging of the hemispherical calibration target showing single-focus images acquired at (a) focus #2 and (b) focus #4. (c) Composite image generated using the images acquired at the five focal depths. For each OCT image and to ease visualization, three ROIs located at specific depths are zoomed in. (d) Lateral maximum intensity projection of the contrast-to-noise ratio (${\mathrm{CNR}}_{S}$) for single-focus and composite images. Focal ranges are separated by four vertical equidistant dotted lines. ROIs are identified by their color-coded rectangles. (e) Quantification of the lateral spatial resolution (σ) in each ROI from OCT images acquired at the five focal depths. Image scales in OCT images are 500  μm.

Figure 4 shows optical coherence tomography (OCT) images and image metrics evaluated in the radial section of the hemispherical calibration target when positioning the focal plane equidistantly from the top (focus #1) to the bottom (focus #5) of the target. In particular, Figs. 4(a) and 4(b) show single-focus OCT images acquired at focus #2 and focus #4, respectively. Figure 4(c) shows the composite image resulting from the combination of focus #2 and focus #4, as well as the three other single-focus images acquired at their specific focal depths. In these OCT images, three ROIs are color-coded and magnified on the right side of each sub-figure. Figure 4(d) shows the ${\mathrm{CNR}}_{S}$ of single-focus and composite images as a function of depth. The five focal ranges are separated by four vertical dotted lines. Except in regions without structures (${\mathrm{CNR}}_{S}<20\;\mathrm{dB}$), ${\mathrm{CNR}}_{S}$ assessed in the composite image is equal to the ${\mathrm{CNR}}_{S}$ assessed in the focal region of each single-focus image. This observation is due to the match between the FWHM of each Gaussian-weighted curve and the distance separating the focal planes. However, local mismatches are also observed around the focal transitions. In the composite image, med(I)=34  dB and $\mathrm{var}(S_{\Omega })=2.4\;\mathrm{dB}$. Maximum gain in contrast reaches 20 dB at the surface of the target. Figure 4(e) shows the depth-dependent lateral spatial resolution of the system (σ) in each ROI and focal plane. Lower values of σ correspond to higher spatial resolutions. The highest spatial resolutions in ROI #1, #2, and #3 are observed at focus #2, #3, and #4, respectively. This result is appropriate because these focal planes are located in these ROIs. Compared with the composite image, σ doubles in single-focus images: from ∼ 6 μm to ∼12  μm in ROI #1 and from ∼4  μm to >8  μm in ROI #2 and ROI #3. In each ROI, the resolution of the composite image (bold black cross) is slightly lower (by less than 1  μm) than the minimal value obtained by one of the single-focus images. These small differences are induced by the weighted averaging process used to generate the composite image. The performance of the fit procedure used to assess σ provided $R^{2}$ values between 0.9947 and 0.9987, which indicates that the error between the fit and the experimental data is low.

### 3.2 *In Situ* Experiments

**Fig. 5:**
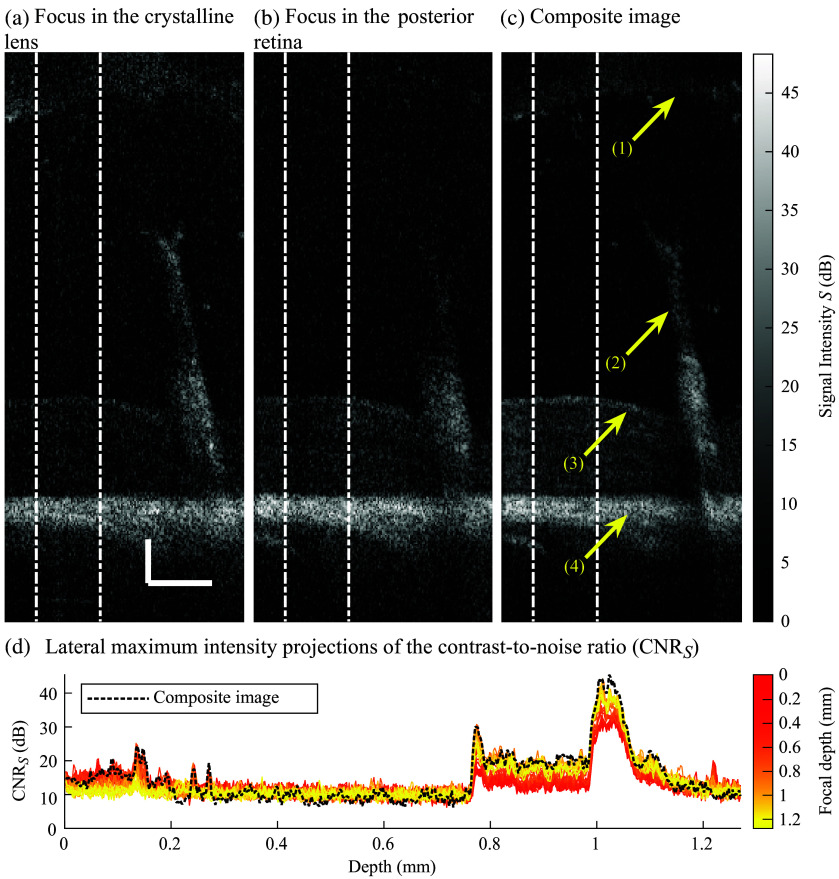
Optical coherence tomography images acquired during *in situ* experiments with the focal plane positioned in the (a) crystalline lens and (b) posterior part of the retina. (c) The composite image of the vitreoretinal vasculature. The arrows indicate (1) the crystalline lens, (2) the hyaloid artery, (3) the retina, and (4) retinal pigment epithelium. (d) Lateral maximum intensity projection of the contrast-to-noise ratio (${\mathrm{CNR}}_{S}$) located between the white dashed-dotted lines along the depth axis and color-coded for the depth of the focal plane. Scale bars indicate 100  μm.

Figures 5(a)–5(b) show OCT images of the mouse vitreoretinal space acquired *in situ* with the focal plan positioned in the crystalline lens and posterior retina, respectively. Figure 5(c) shows the composite image generated with the 100 coplanar B-scans. The arrows indicate (1) the crystalline lens, (2) the hyaloid artery, (3) the retina, and (4) RPE. The hyaloid system is partially described in the posterior part of the vitreous with disconnected vessels due to the 2D nature of the image. Figure 5(d) shows the lateral MIP of the ${\mathrm{CNR}}_{S}$ located between the white dashed-dotted lines of each OCT image in Figs. 5(a)–5(c). The composite ${\mathrm{CNR}}_{S}$ follows the ${\mathrm{CNR}}_{S}$ from the optimal single-focus images acquired in the crystalline lens and retina. The contrast in the composite image shows some variations between 0.2 and 0.75 mm depth, a region in the vitreous without reflective structures except for punctual hyaloid remnants (detached structures generated during hyaloid regression). Compared with single-focus images, the ${\mathrm{CNR}}_{S}$ of the composite image is improved by 8 to 10 dB at the lens-to-vitreous interface (∼ 0.18  mm deep) and in the posterior retina, including the RPE and choroid (∼[0.8 to 1.1] mm) deep). The gains in signal intensity are proportional to the gains in ${\mathrm{CNR}}_{S}$.

### 3.3 *In Vivo* Experiments

**Fig. 6:**
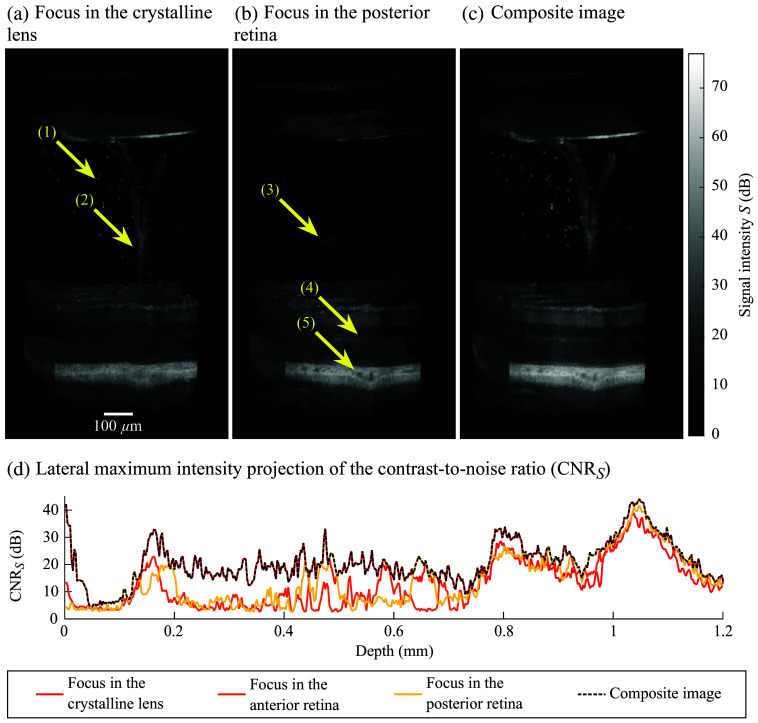
Optical coherence tomography (OCT) images acquired during *in vivo* experiments with the focal plane positioned in the (a) crystalline lens and (b) posterior retina. The arrows indicate (1) hyaloid remnants, (2) hyaloid vessels, (3) an image artifact, (4) the outer plexiform layer, and (5) choroidal structures. (c) Composite image of the vitreoretinal vasculature. (d) Lateral maximum intensity projection of the contrast-to-noise ratio (${\mathrm{CNR}}_{S}$) as a function of depth for single-focus OCT images positioned in the crystalline lens (red), anterior (orange), and posterior (yellow) retina, overlapped with the composite image signal (black dashed line). OCT image voxels are isotropic (scale bar is 100  μm).

Figure 6 shows 3D views of an OCT C-scan acquired *in vivo* in a P14 mouse with focal planes located in Fig. 6(a) vitreous proximal to the crystalline lens and Fig. 6(b) posterior retina. Figure 6(c) shows the composite image generated from volumes [Figs. 6(a) and 6(b)], as well as a volume acquired with the focal plane located in the anterior retina. The arrows indicate (1) hyaloid remnants and (2) hyaloid vessels. These structures are invisible when the focal range is not located in the vitreous [Fig. 6(b)]. The arrow (3) indicates a structure that appears as an artifact. The other arrows indicate the (4) outer plexiform layer and (5) choroidal structures, which are well defined when the image is acquired with the focus located in the posterior retina. Figure 6(d) shows the lateral MIP of the ${\mathrm{CNR}}_{S}$ of single-focus images acquired with focal planes located in the crystalline lens and in the anterior and posterior retinas, as well as for the composite image. The composite ${\mathrm{CNR}}_{S}$ shows the contributions of both single-focus images acquired in the crystalline lens (depth <0.7  mm) and in the retina (depth >0.7  mm), which allows visualization of the vitreoretinal vasculature in full depth. Compared with single-focus images, increases in composite ${\mathrm{CNR}}_{S}$ are observed in the hyaloid vessels [15 dB, between ∼[0.15 and 0.7] mm] and the retina (5 dB, between ∼[0.8 and 1.1] mm). The gains in signal intensity are proportional to the gains in ${\mathrm{CNR}}_{S}$.

## 4 Discussion

In this work, a multifocal OCT approach and a registration method were developed to image the vitreoretinal vasculature of the mouse eye. The optical system was calibrated using a custom-made hemispherical target and a method was presented to quantify signal intensity, image contrast, and lateral spatial resolution. The feasibility of the technique was demonstrated through *in situ* and *in vivo* experiments. For these experiments, a composite image generated from single-focus images showed higher signal intensity and image contrast, and a finer, uniform resolution compared with single-focus images.

In each experiment, the combination of OCT signals acquired at multiple focal planes allowed the generation of a composite image characterized by a more uniform signal intensity and image contrast throughout the field of view compared with single-focus images. Each single-focus image contributed to a higher signal intensity and contrast proximal to its focal plane. This technique was previously used to image cerebral blood flow in deep cortical layers in mice and presents similarities with the use of dynamic focus in OCT.^18^^,^^20^ Our results suggest that this multifocal technique is useful for imaging the vitreoretinal vasculature in full depth and could be used for other structures that require a large imaging field of view.

For experiments using the calibration target, regions located around the focal plane showed higher signal intensity and sharper edge contrast than the background. In addition, better lateral spatial resolution was observed with a decrease in the distance between the focal plane and the structure. This relationship was observed for the three ROIs and highlights the performance of the technique under controlled and quantifiable conditions.

The lateral resolution was quantified to be as low as 4 to 6  μm in the calibration target. In animal experiments, the multifocal approach improved signal intensity, image contrast, and lateral spatial resolution, particularly for small structures such as fine hyaloid vessels and remnants. These structures were not visible when using only a deep focal plane located in the posterior retina. These results suggest that our technique could also be used to image small structures such as mouse retinal capillaries.

In the retina, the gains in signal intensity and image contrast in the composite image appeared more marginal than in the hyaloid vasculature. This observation may be due to the anatomical structure of the retina, which is composed of several layers of specific cells that are not as reflective as the RPE. Imaging anatomical tissues with high and homogeneous extinction coefficients could have possibly resulted in greater gains in signal intensity and image contrast.^18^^,^^20^ Although signal intensity presented in previous studies appeared to be higher, it was not quantified.^2^^,^^10^^,^^11^ This higher signal intensity from previous studies may also be due to the use of OCT systems emitting light with a central wavelength at 840, 850, and 1048 nm, which are more suitable for ocular imaging than our instrumentation.

*In situ* and *in vivo* images were acquired in healthy P14 mice. Because hyaloid regression typically begins before P14,^35^ imaging younger animals could have allowed imaging a larger group of hyaloid vessels. Although these *in vivo* experiments can be challenging, it is possible to image the retina as early as P7.^30^ Imaging animals with pathological conditions, such as persistent hyperplastic primary vitreous, could also have allowed imaging a larger hyaloid vasculature.^11^

This work has limitations. Although the animal eye and the glass sphere shared the same function of focusing the light onto the retina/target, the focal shift was different in the two environments. Thus, the purpose of the calibration target was only to show the capabilities of the lens in the lateral scanning scheme. For angular scanning, a series of B-scans was performed as described above. A gradual loss of signal intensity was observed with increasing depth (signal roll-off), even if a multifocal approach was used throughout the field of view. However, for a small organ such as the mouse eye, signal roll-off was limited to ∼1  dB.^36^ The signal roll-off also affected the noise floor level used in the scaling process. Although this effect was minimal in regions of low signal intensity, it affected individual MIPs similarly. Using an MIP to quantify the signal intensity and image contrast generated a nonzero noise level in regions without OCT reflectance. This anomaly was induced by the remaining noise signal after subtracting the noise floor. In addition, the two image registration methods used in animal experiments flattened the retina. However, this consequence of image registration facilitated the generation of a composite image obtained by combining individual images that were affected by a different level of motion. Fast motion, such as respiration rate, and slow motion, such as drift or bulk motion of the animal, affected the registration of single-focus OCT images. Removing highly contaminated images was necessary to avoid registration failure and the generation of the composite image. Furthermore, erratic bulk movements prevented the use of angiography-suited OCT algorithms,^37^ which would potentially improve the description of hyaloid vessels. Once these limitations are overcome, future works include a longitudinal study to monitor *in vivo* the hyaloid regression/persistence and concurrent retinal changes in the context of physiological development and disease.

## 5 Conclusion

This work demonstrated the potential of multifocal OCT imaging to study the vitreoretinal vasculature in full depth. A tunable lens was used to acquire multiple images of the hyaloid and retinal vasculature at specific focal depths. By combining the registered images, the signal intensity and image contrast became more uniform throughout the entire field of view, allowing visualization of both the retina and the hyaloid vessels. The main advantage of this technique remains the better lateral spatial resolution over the entire field of view, quantified using a custom-made calibration target. This technique shows promising abilities to study the mouse vitreoretinal vasculature during eye development and disease.

## Supplementary Material

10.1117/1.JBO.30.11.116002.s01

## Data Availability

Imaging datasets acquired in this work are not publicly available at this time, but may be shared by the authors upon reasonable request.
