# Supplementary material for: Multifocal optical coherence tomography of the mouse eye to image the vitreoretinal vasculature in full depth
Source: J Biomed Opt. 2025 Nov 3;30(11):116002. doi: 10.1117/1.JBO.30.11.116002 (PMC12582522; doi:10.1117/1.JBO.30.11.116002)
Supplement: Supplementary file 1 [file JBO_030_116002_SD001.pdf]

Multifocal optical coherence tomography of the mouse eye to image the vitreoretinal vasculature in full depth

Simon Brais-Brunet[a,b], **Raphaël Maltais-Tariant**[c], **Caroline Boudoux**[b,c,d], **Mathieu Dehaes**[a,b,*]
[a]Institute of Biomedical Engineering, Université de Montréal, Montreal, Quebec, Canada
[b]Centre de Recherche Azrieli du CHU Sainte-Justine, Montreal, Quebec, Canada
[c]Department of Engineering Physics, Polytechnique Montréal, Montreal, Quebec, Canada
[d]Castor Optics, Saint-Laurent, Quebec, Canada
[e]Department of Radiology, Radio-oncology and Nuclear Medicine, Université de Montréal, Montreal, Quebec, Canada

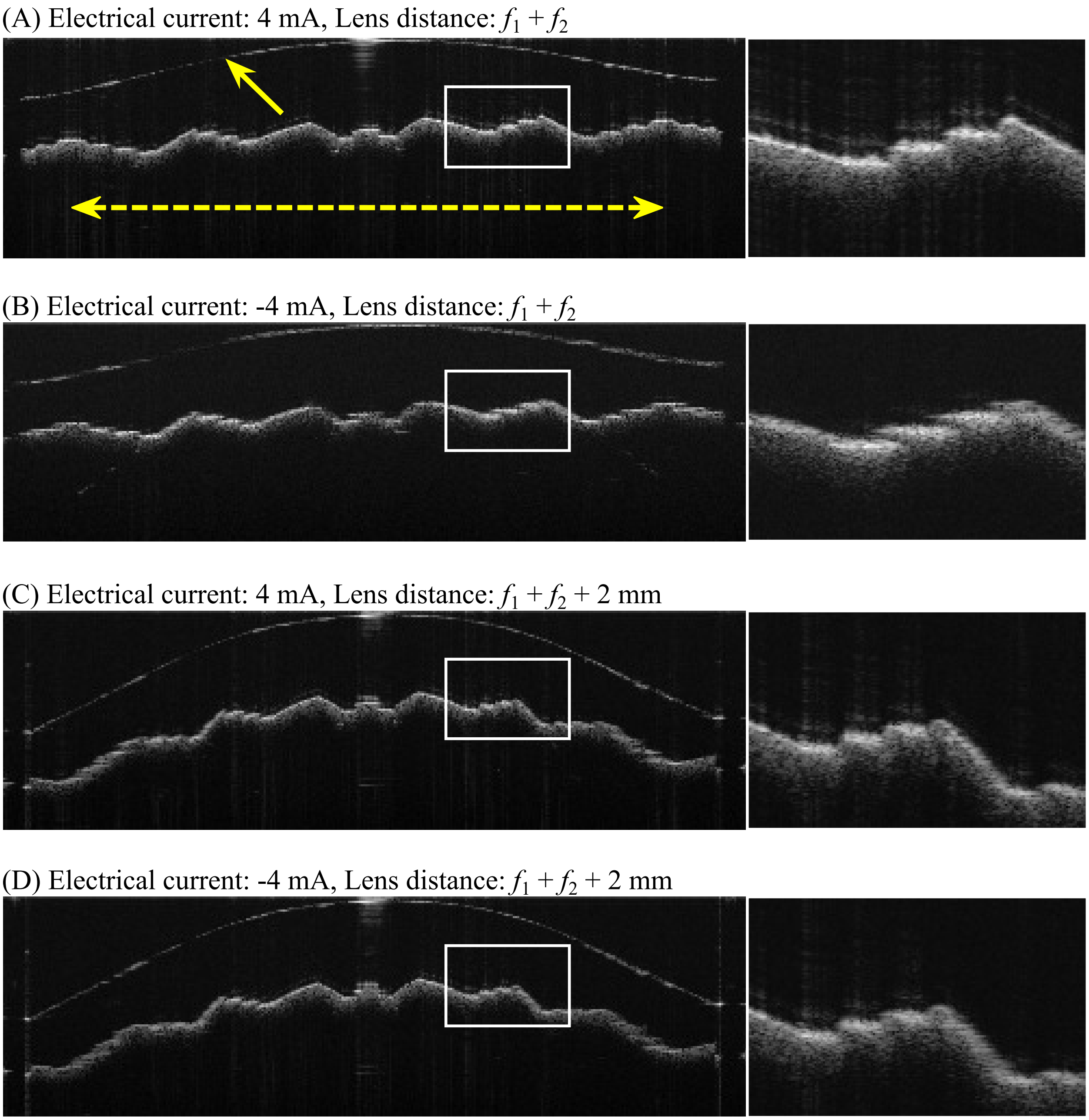

Fig. S1 Field of view distortions: Optical coherence tomography images acquired in the calibration target using the glass sphere (see yellow arrow) and an electrical current of ± 4 mA when using a lens distance of (A, B) 165 mm (focal lengths $f_1 + f_2$) and (C, D) 167 mm ($f_1 + f_2$ + an offset of 2 mm). Engraved structures located in the white boxes are magnified in the right sub-figures. An angular scanning scheme was used. The yellow dashed double arrow shows the spatial range of a scan of 90° or approximately 3.927 mm.

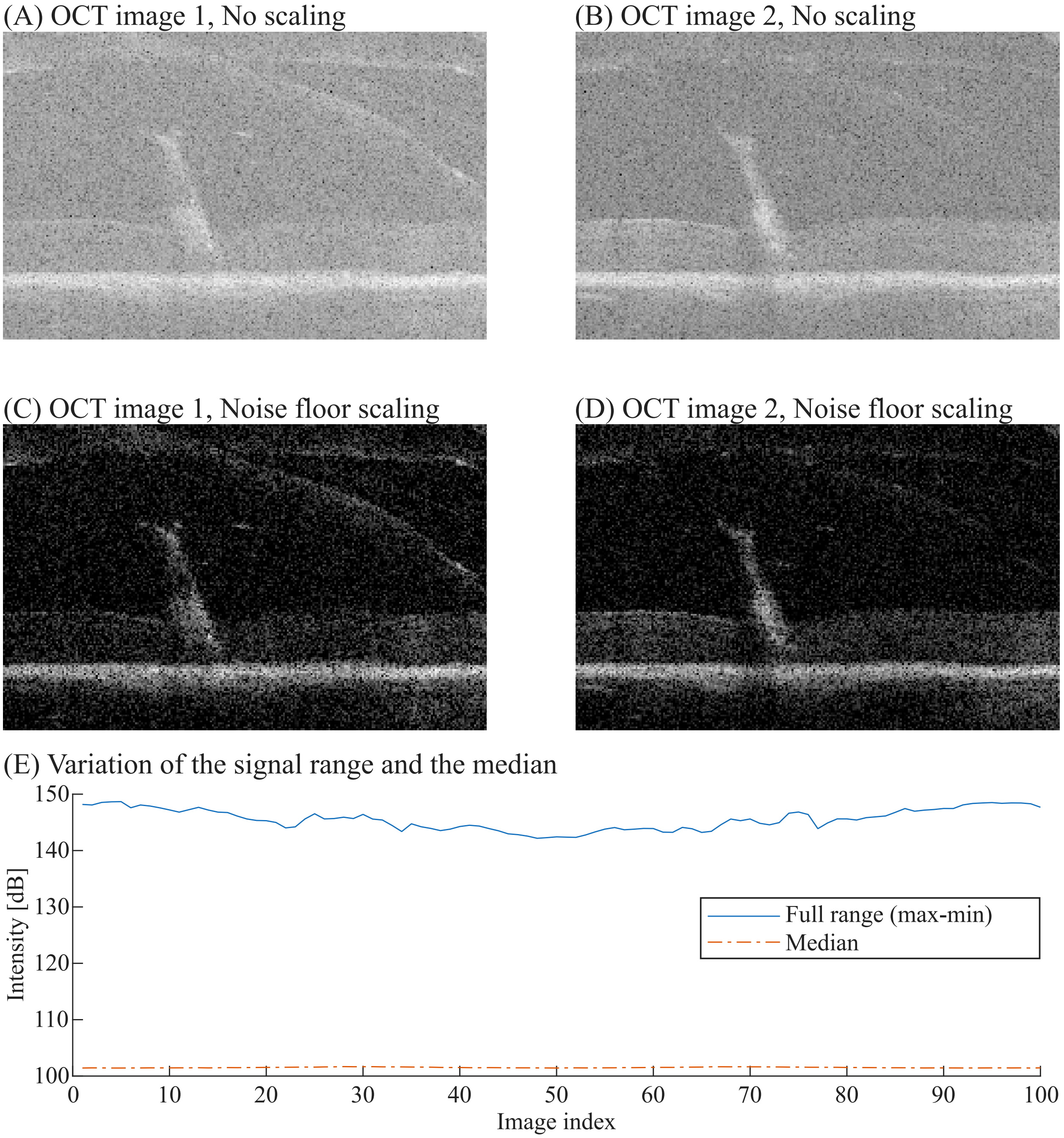

Fig. S2 Effect of the noise floor scaling on two random optical coherence tomography (OCT) images. Without noise floor scaling (A and B), images were scaled by the full range of the image (max-min values). In these cases, noise floor contributes to the signal and the overall brightness of the image can be affected by the amplitude of this range. The noise floor scaling by the median of the raw signal (C and D) removes the contribution of the noise floor, improving the contrast. (E) The noise floor scaling flattened the variation in brightness as the scaling factor remained stable for all pixels as opposed to the full range value.
